# Navigating the Online World of Lifestyle Health Information: Qualitative Study With Adolescents

**DOI:** 10.2196/35165

**Published:** 2022-02-11

**Authors:** Rebecca Raeside, Si Si Jia, Julie Redfern, Stephanie R Partridge

**Affiliations:** 1 Engagement and Co-Design Research Hub Faculty of Medicine and Health University of Sydney Westmead Australia; 2 The George Institute for Global Health University of New South Wales Sydney Australia; 3 Prevention Research Collaboration Faculty of Medicine and Health University of Sydney Sydney Australia

**Keywords:** adolescents, chronic disease prevention, websites, social media, smartphone applications

## Abstract

**Background:**

Adolescence is a critical life stage characterized by an interplay of biological, social, and environmental factors. Such factors influence lifestyle health-related trajectories, including dietary behaviors, physical activity levels, body weight, and sleep. Generation Z (born 1995-2015) is the most internet-dependent and technologically savvy generation in history with increasing rates of smartphone ownership across high- and low-income countries. Gaps exist in understanding what online platforms adolescents are using and barriers and facilitators of these platforms to seek lifestyle health information.

**Objective:**

We evaluated adolescents’ perceptions on the use of contemporary digital platforms (websites, social media platforms, smartphone apps) to seek lifestyle heath information or advice.

**Methods:**

Virtual focus groups were held via Zoom teleconference between July 2021 and August 2021. Eligible participants were 13 years to 18 years old, were living in Australia, and had searched for online lifestyle health information in the previous 3 months. For this study, lifestyle health information referred to key behaviors and risk factors for chronic disease, namely, diet, physical activity, weight management, and sleep. Participants were recruited through an existing database of research participants and networks of the research team. Focus groups were analyzed using the framework approach, in which data are systematically searched to recognize patterns in the data and manage, analyze, and identify themes. Focus group audio files were transcribed verbatim and independently coded by 2 researchers (RR, SSJ). Through an iterative, reflexive process, a final coding matrix was agreed on by all researchers and used to thematically analyze the data.

**Results:**

We held 5 focus groups (n=32; mean age: 16.3 [SD 1.4] years; 18/32, 56% female; 13/32, 41% spoke language other than English at home). Thematic analysis revealed participants searched for information both actively (eg, on Google or YouTube) and passively (eg, scrolling social media and using existing apps preloaded to their smartphone such as Apple Health, Samsung Health, or Google Fit apps). Participants identified that the most helpful information was well-presented in terms of aesthetic appeal and layout and came from a credible and reliable source (eg, any sponsorships disclosed), and they expressed the need for the information to be relatable. Mixed views were reported for the application of lifestyle health information found online. Some participants reported behavior change, while others noted that certain advice was hard to maintain and incorporate into their lifestyle.

**Conclusions:**

This study highlights the abundance and complexity of lifestyle health information online for adolescents. Adolescents in the digital age seek access to information that is appealing, credible, relevant, and actionable for lifestyle health behaviors. To appeal to needs of adolescents, future interventions for adolescents relating to lifestyle health must consider co-design methodological approaches. Furthermore, the regulation of lifestyle health information available online warrants further investigation.

## Introduction

Today’s adolescents, defined by the World Health Organization (WHO) as aged 10 years to 19 years, make up 16% of the global population [1]. Adolescence is a critical life stage during which a complex interplay of biological, social, and environmental factors determines the trajectory of lifestyle health behaviors into adulthood [2]. Lifestyle health behaviors and risk factors that are of great importance during adolescence are diet, physical activity, weight management, mental health, and sleep hygiene, as they are predictors of adverse health outcomes in adulthood, such as obesity and cardiovascular disease [3]. Globally, most adolescents do not meet diet or physical activity guidelines [4,5], and there has been a dramatic increase in the prevalence of overweight and obesity, jumping from 4% to over 18% in the last 40 years [6]. In Australia, very few adolescents meet guidelines for diet and physical activity [7,8], and adolescents do not get enough sleep on school nights [9]. Adolescence is an opportunistic window for establishing good lifestyle health behaviors [10]. Despite this, research priorities during adolescence are often focused on reducing other high-risk behaviors such as suicides, substance use, and sexual activity, with limited attention given to research that effectively harnesses digital technologies to target prevention of chronic diseases through lifestyle risk factor management [11,12].

Adolescents are known as “digital natives” as they have been born into a ubiquitous digital environment [13], which has grown exponentially in the last 20 years. In Australia, 94% of adolescents own a mobile phone, 95% are accessing the internet daily, and they use an average of 4 different social media platforms [14]. Previous research has shown that adolescents frequently turn to online sources such as internet websites and social media for lifestyle health information [15]. A national US survey found that adolescents are primarily looking at diet and fitness information online, with more trust placed on the internet than social media [16]. Furthermore, studies have explored how adolescents search for and appraise online health information and the extent to which they trust this information [17,18]. Furthermore, there is a constant expansion in the variety of digital platforms, including the uprise of contemporary platforms such as TikTok and Discord. As such, the current evidence base exploring the use of digital platforms to obtain information on lifestyle health behaviors is outdated. Contemporary digital platforms are a highly appealing and easily accessible way for adolescents to obtain lifestyle health information, given the increasing rates of smartphone ownership and their widespread use among adolescents for the pursuit of lifestyle health information.

As the digital health space is growing, gaps exist in our understanding of what contemporary digital platforms adolescents are using to seek this information and the barriers and facilitators of obtaining lifestyle health information on these platforms. Understanding the barriers and facilitators is crucial for governments, health organizations, researchers, and policy makers to be able to deliver appealing and effective lifestyle health promotion and support adolescents with management of chronic disease risk factors. Therefore, the aim of this study was to explore adolescent perceptions of obtaining information or advice related to lifestyle health from contemporary digital platforms.

## Methods

This study adhered to the consolidated criteria for reporting qualitative research (COREQ) guidelines for reporting qualitative research (Multimedia Appendix 1) [19]. The study protocol was approved by the University of Sydney Human Research Ethics Committee (approval number 2020/613), and participants gave informed e-consent prior to participation.

### Participants

Participants who were eligible to take part in the focus groups were aged 13 years to 18 years (inclusive). The WHO defines adolescents as 10 years to 19 years old; however, the range of 13 years to 18 years was selected to coincide with the age range of secondary education in Australia, which is a common setting for health promotion interventions by governments. Further eligibility criteria included living in Australia and having had accessed lifestyle health information online at least once in the previous 3 months. For this study, lifestyle health information referred to key behaviors and risk factors for chronic disease, namely, diet, physical activity, weight management, and sleep.

### Recruitment

Participants were recruited through an existing database from a previous cross-sectional survey (Digitalize Study) [20] and known networks to the research team. The Digitalize Study was a cross-sectional survey to find out how young people (13-18 years old) search for lifestyle health information online including which digital platforms were most used, perceived helpfulness of information on digital platforms, helpfulness for positive behavior changes, and the quality of platforms’ health information. Email invitations were sent with a link to the participant information sheet. All prospective participants read the participant information sheet online, provided informed e-consent, and were directed to an online survey to indicate demographic characteristics (age, gender, postcode, and language spoken at home) and how often they searched for lifestyle health information online in the previous 3 months. A 3-month time frame was chosen so that participants had up-to-date knowledge of lifestyle health information on these digital platforms. If participants had not accessed lifestyle health information in the previous 3 months, they were not able to complete the survey and therefore were not contacted to take part in the focus groups. All eligible participants were contacted via text message to confirm date and time of focus group and were emailed the secure teleconference link.

### Data Collection

A semistructured discussion guide was developed by the research team based on the outcomes of the Digitalize study [20] to further explore the perceptions of obtaining and using lifestyle health information online. To assess whether the focus group questions were easy to understand and acceptable, the interview guide was piloted with 2 youth advisors who currently work with the research team. The discussion guide is provided as supplementary material (Multimedia Appendix 2).

One researcher (RR) gave a brief overview of the discussion at the commencement of the focus groups. Participants were asked about where they accessed health information online (internet websites, social media platforms, and smartphone apps), why they used online sources, what type of content they found most engaging, and any potential changes to their lifestyle behaviors as a result of applying the information they obtained online. Based off their responses, the 2 platforms of most interest to the group were discussed in more detail. Questions explored how they searched for information on these online sources, what made these sources most and least appealing, and how they judged the reliability and usefulness of the information they found.

The focus groups were conducted by 2 researchers (RR, SSJ) via videoconferencing (Zoom Video Communications Inc, San Jose, CA) at a time convenient for participants. The focus groups were led by RR, and SSJ took detailed notes for each session. Each focus group took approximately 45 minutes to complete. RR has training and previous experience in conducting focus groups and semistructured interviews. Focus groups were recorded and transcribed verbatim into Microsoft Word (Version 16.54, Microsoft 365, Microsoft Corp, Redmond, WA) by RR. Recruitment of participants for focus groups ceased when thematic saturation was reached. Participants were not contacted for further focus groups or validation of transcripts. Each participant was provided with an Aus $20 (US $14.26) gift voucher for participation.

### Data Analysis

The framework approach was used to analyze qualitative data [21], where data are systematically searched to recognize patterns in the data and manage, analyze, and identify themes. On completion of focus groups, RR and SSJ familiarized themselves with the data and undertook thematic analysis independently. RR and SSJ developed coding labels relevant to the research question and identified emergent themes. After systematically coding all the transcripts, the research team (RR, SSJ, and SRP) discussed themes that were further developed through an iterative and reflexive process. Consensus on final themes were developed and agreed on by all researchers. Qualitative data analysis was performed using NVivo 12 (12.2.0).

## Results

### Participant Characteristics

Focus group attendance was confirmed by 37 participants; 5 participants did not attend the focus groups without providing a reason, leaving a total sample of 32. Participant characteristics are reported in Table 1. Participants had a mean age of 16.3 years. From the study sample, 56% (18/32) of participants identified as female, with most participants residing in New South Wales (22/32, 69%). Two-fifths (13/32, 41%) spoke a language other than English at home. Participants varied in their frequency of accessing health information online with over one-third accessing health information online 1 to 2 times a month (12/32, 38%).

**Table 1:** Focus group participant characteristics (n=32).

Participant characteristics			Results
**Age (years), n (%)**			
	13-14	4 (13)	
	15-16	11 (34)	
	17-18	17 (53)	
Age (years), mean (SD)			16.3 (1.4)
**Gender, n (%)**			
	Male	13 (41)	
	Female	18 (56)	
	Prefer not to say	1 (3)	
**Residential state in Australia, n (%)**			
	New South Wales	22 (69)	
	Victoria	6 (19)	
	Western Australia	4 (13)	
**Language spoken at home, n (%)**			
	English only	19 (59)	
	>1 other languages spoken	13 (41)	
**Frequency of accessing lifestyle health information online, n (%)**			
	1-2 times a month	12 (38)	
	Once a week	7 (22)	
	A few times a week	8 (25)	
	Once a day	1 (3)	
	More than once a day	4 (13)	

### Themes

#### Overall Findings

**Figure 1:**
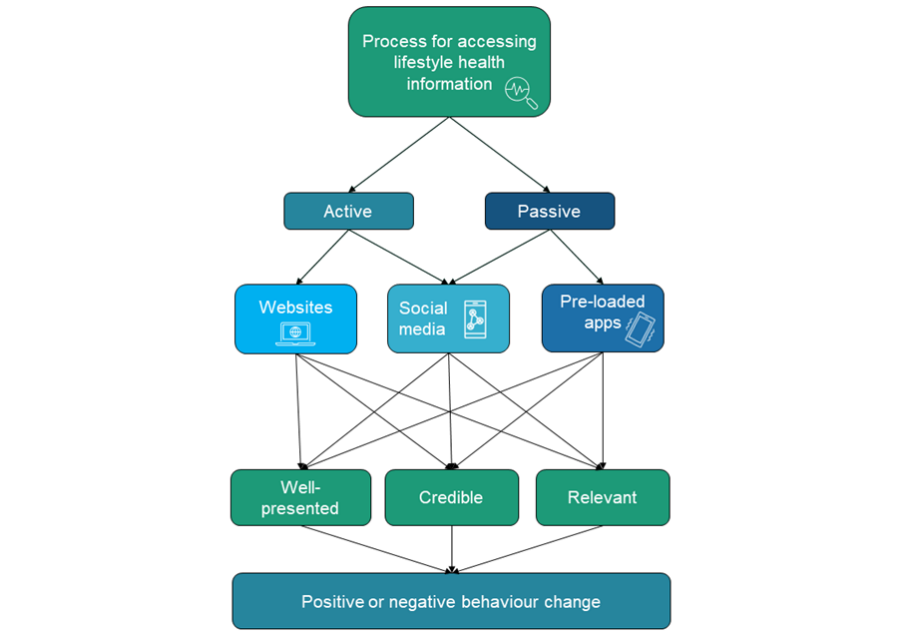
Conceptualization of emergent themes related to participants obtaining lifestyle health information across contemporary digital platforms.

Thematic analysis identified a complex interplay of 5 main themes relating to obtaining lifestyle health information across contemporary digital platforms (Figure 1). These 5 themes included the processes of accessing lifestyle health information online, the presentation of lifestyle health information online, the importance of credible and reliable information, having information relevant to adolescents, and perceived behavior changes from application of lifestyle health information found online. These themes emerged across the 3 digital platforms that were discussed in-depth (internet websites, social media platforms, and smartphone applications). Across different digital platforms, there were distinct similarities and differences, which are explored in detail in each theme in the next sections. Despite attempting to ascertain which type of lifestyle health content (ie, diet, physical activity, weight management, or sleep) would be most engaging to adolescents, all 5 focus groups did not have a clear emerging theme, with all aspects of lifestyle health discussed. The 5 emergent themes are discussed in detail in the following sections.

#### Theme 1: Processes for Accessing Lifestyle Health Information Online

Adolescents identified internet websites and social media platforms as the top 2 sources for lifestyle health information online. However, they described the process for accessing information from these 2 sources differently. On digital platforms such as Google and YouTube, participants described actively searching for information of interest to them. Google searching for information was reported by most participants, who stated that they would only visit the first few websites that appeared in the search results. Websites that were commonly frequented by participants for health information were government-based websites and blogs. Some participants searched on YouTube for health information, especially relating to exercise and recipes.

I think that I would probably go and just do a google search honestly, and then whatever would come up there is what I would do. I don’t think I’d refer to a specific website.

FG2, 18M

For me, it’s like on Google, I usually get my information from the first like 10 or 8 websites or something, like the more backdated it is like in the second or third page, I feel like it would be less relevant to me.

FG3, 17M

Accessing health information on social media platforms was described differently as participants would passively receive information that appeared in their feed (from people who they chose to follow) or by scrolling through explore pages. Participants also identified that the information that they came across on social media platforms may have been targeted to them due to algorithms used by these platforms.

...it’s not really like me actively searching up on Instagram; it’s more me following like a few organizations and people like government, a few athletes, and physios and things like that.

FG4, 16F

I don’t search that much for things cause things I [want to] know, they usually just come to me on my feed because you know TikTok and Instagram, they are really customized...

FG5, 13F

Smartphone applications were also identified by some participants as being used to access lifestyle health information. Similarly, for smartphone applications, participants mostly reported using applications that were already available on their smartphone (eg, Apple Health, Samsung Health, Google Fit) rather than searching app stores for new applications. When exploring reasons behind app usage, cost was a major factor, which supports this finding of using apps that are readily available to them.

I’ve [got to] say, I also use the standard health app on my phone, which is actually quite helpful cause I can track how much I exercise, how much I run, also other health-related things.

FG4, 16F

As I said, my phone already came with the app, so I didn’t look for it.

FG4, 17M

I wouldn’t spend money on an app to tell me all those things cause (sic) I can just find it on the internet.

FG4, 16F

#### Theme 2: Presentation of Lifestyle Health Information Online

Participants placed a large emphasis on the importance of how lifestyle health information is presented and organized for them online to be able to easily read and to interpret the information. This included the importance of white space and the use of dot points and subheadings on websites that would make them “easy to navigate” [FG4, 17F]. They reported that, if websites were not laid out logically or in an aesthetically appealing manner, they would often look elsewhere for the same information.

I’d say like organized well, like I don’t want it to have all these clunks of information that is not actually relevant to what I’m looking for...and also like, you know, have lots of like white space on the page so it’s easier to interpret and like dot points.

FG1, 14M

If the layout is really bad or like the words are all really close together, it just overwhelming to look at the website so I would be way more likely to click off.

FG2, 18F

Another subtheme that emerged was the importance of the quality of the content. This was reported both in terms of the actual information that was being presented and the production quality of videos or posts on social media. When information was presented in a way that appeared to be high quality and aesthetically appealing, most participants reported that they found it more credible. On social media, the production of posts or videos needed to be of good quality for them to follow that person and to “dig deeper” [FG5, 13F] into the account.

It’s kind of a mix of both because if I like their content, I’ll probably follow them, but if they have put like no effort into their page, it kind of like throws me off because I’m the type to like aesthetic-looking stuff.

FG5, 14F

I think the information they put out is important, but I think I’m less likely to actually follow them if the production is not as good as other people.

FG5, 15M

#### Theme 3: Credible and Reliable Lifestyle Health Information Online

Nearly all participants identified that they assessed whether the health information online was credible or reliable and this was achieved differently depending on the online source. For websites, participants reported looking if the information was referenced or included a bibliography. It was also recognized that participants mostly trusted government websites and well-known health organizations, but when it came to other websites, that they would “cross check” [FG1, 18F] to see whether there was a scientific backing to the information presented. By verifying this information with other sources, this demonstrates an awareness of where credible information originates.

I think it is based off your source, but most of the government stuff is pretty good.

FG1, 17F

I look for research papers, and if it’s a government website or any sort of university and if it’s a newsletter or something like that, then I try to check it twice with something else.

FG3, 17M

I think usually when you look at information, you can just kind of judge just by the way that they’re putting forward the information and also just to cross check just Google to see whether their information matches up what other people are saying majority of the time.

FG1, 18F

On social media platforms, participants identified multiple ways they assessed whether what they saw was credible or reliable. First, participants emphasized the importance of having a person behind the account with their credentials clearly stated. Many participants also stated that if they had a blue tick, meaning that the account was verified, this would also increase their credibility. Furthermore, the follower count of the social media account was also seen to increase reliability, with more followers making them more reliable.

I think knowing more about the person behind it is useful because sometimes you will find information and you can tell that it is like really biased. It’s information for sure, but it’s what they want you to know, it’s not really always true. So, if they could say like who they are and where they got the information, it would be a lot more trustworthy.

FG2, 17F

Well, if I see like a tick...like that blue tick, that they’re like professional, I guess, and also like if I see that they have a lot of followers, I don’t know sometimes, some organizations they have [been] verified by the NSW government and things like that.

FG4, 16F

Nearly all participants were acutely aware of sponsorships and advertising online. On social media, influencers are often paid to promote certain products or services. For most participants, if an account was constantly promoting one product, this would be a deterrent to trusting the information that they portrayed and was seen to be “off putting” [FG2, 18F]. Participants also understood that this was a way in which these accounts generated income.

I was just going to say that I think the content really matters and whether or not they're putting it out for the right reasons. I see, like sometimes, influencers, is that they are just kind of putting up information because they’re getting paid to, you know, to advertise for those things.

FG1, 18F

I think if they are constantly advocating for like 1 idea or like 1 diet or, you know, 1 product or something like that, kind of puts you off because it shows that it’s like, it’s not genuine, it doesn’t show other sides, and then you’re kind of being biased.

FG2, 17F

If it’s really pushing it and especially like Instagram and social media where it’s just dedicated to that thing, to that endorsement, it’s a little more off putting. But you know it’s how it goes, they have to do it, so if I’m good with the content that they provide otherwise, then it’s OK.

FG3, 17M

Regarding advertising on websites, participants reported the type of advertisements were important. If the advertisement was unrelated to the website itself, then the website would be seen to be unreliable. However, having advertisements in general was seen to be acceptable, and when the information was referenced correctly, they would still consider the website to be reliable.

Definitely the type of ads, like if I see something that I’m like no that’s not right, I’ll probably get off the website as soon as like I see it.

FG5, 14F

Just the ads doesn’t really deduct from the website for me, cause for me, it’s like if they cross reference it with at least one or two sites, like the first three sites no matter how un-user friendly they are if they say the same thing, then I just take that away from it.

FG3, 17M

Another subtheme that was identified was that some participants assessed other people’s comments on social media posts to see other opinions about whether the information that was presented was reliable. Likewise, when assessing mobile applications that they would potentially download, some participants looked at reviews on app stores to see whether other people thought the app was helpful for them.

I do go to the comments every single video or pretty much that I’m interested in to see what other people think about it, not for information purposes but sort of to see what other people’s opinion on that particular post is.

FG5, 15M

I would also definitely look at the reviews and not just what the reviews say but the amount of the reviews, how many people access the app, how many people say this or that, that kind of thing.

FG4, 16F

#### Theme 4: Lifestyle Health Information Relevant to Adolescents

Most participants reported that searching for health information online was convenient. Participants reported online information as being easy to access, readily available, and regularly updated. Also, many participants reported that they accessed lifestyle health information from a variety of sources to compare the information themselves rather than seeing a health professional, which takes time and money and they may not provide the extent of information that can be found online.

Mainly because it’s very accessible and just easy to access, very like fast, just search it up, and you basically have an answer. Um, also, I guess, you can find people who are going through similar situations, like same age group just people that are, who you can relate to, and I think that’s generally where you can get a lot of advice from.

FG4, 16F

I think because it's always like updated, whereas if you've got like an out-of-date leaflet or something, it might not be relevant.

FG1, 18F

I definitely think it’s easier to look it up online because then you just get a bigger range of answers as well like if you go to a doctor, they normally just give you one straight answer.

FG2, 18F

When referring to social media specifically, many participants reported following accounts that were relatable. For an account to be relatable, they had to engage frequently with their followers in terms of posts or stories and have lifestyle health information and advice that would be easy to implement into their own lives. Also, it was recognized that the person behind the account was important in terms of relatability, due to a sense of familiarity. This did depend on the size of their existing following, with smaller accounts being favored as they were more likely to respond and engage with followers, whereas larger accounts were viewed as more reliable.

I’ll generally follow accounts that like I can relate to so I can kind of use their posts in my own life.

FG2, 18F

You can also find people who are in the same position as you like, for example people who are the same age...and it’s really easy to see if their lifestyle, like you can take something from that so that’s definitely been really helpful for me.

FG4, 16F

I feel like you can, you know, you can build a better relationship and get familiar with what they do on social media and if they are also influencing you in fitness, etc, you follow that.

FG3, 17M

Something like lifestyle, for example, I definitely go for the people who have less followers, if it’s specifically like workout videos I tend to go for the big names like Chloe Ting or those sort of ones.

FG3, 17F

Websites were often reported by participants as being too generalized and therefore were sometimes viewed as unrelatable and unhelpful for obtaining health information. Some participants identified that the information may not be specifically directed toward young people or in line with what they were wanting to achieve in terms of their personal goals. It was also identified that they had visited websites that provided a large variety of information but not enough detail; therefore, they were unable to make a judgement of whether that information would be relevant to them.

I think sort of just having information that is like more relevant to you. I know that a lot of the sort of diet information that I find on the internet, it’s more sort of geared towards adults.

FG3, 17F

For just general health and stuff, I find the government websites pretty lackluster, like they kind of just go through the motions and give the minimum information, so like, for what I’m interested in with training, the government websites aren’t that great.

FG5, 15M

Sometimes, it’s just like you’re not there for a really wordy essay, for example, like you guys are professionals but please make it understandable for us, you know, you’re not trying to show off your skills, you’re trying to provide information that is useful and understandable.

FG3, 16M

#### Theme 5: Perceived Behavior Changes Based on Online Lifestyle Health Information

When regarding behavior change from online health information, participants reported a mixed variety of personal effectiveness. Some participants reported making changes based on information that they had seen online, including dietary changes such as restricting calories and physical activity changes including trying specific workouts that were not effective for them and subsequently viewed these behavioral changes negatively. As the behavioral changes were viewed undesirably, the changes were not sustained long term. Contrary to this, other participants reported making changes such as intermittent fasting and increasing total sleep time, which were viewed by participants as positive changes. It is important to note that, for some lifestyle changes, some of the participants viewed these positively, and others viewed them negatively, demonstrating the complexities of lifestyle health information and how individual preference also plays an important role. Furthermore, some participants outlined desire to make changes; however, the information that they found was “too hard to integrate” [FG4, 17F] or “doesn’t last a very long time” [FG4, 16F].

I saw something online about like how much you should be eating...a day, and I severely restricted my like caloric intake, and I noticed, like, straight away that that in it had an impact.

FG3, 17F

When I found this out about intermittent fasting, it just sort of like suited me better than what the government guidelines tell you.

FG1, 18F

I googled how much sleep someone of my age should be getting, and it turns out it was a lot more...than what I was getting, so I try to go to bed a bit earlier and try to wake up a bit later each day.

FG2, 14F

Additional quotes for all themes are listed in Textbox 1.

**Quotes illustrating participants’ experiences related to obtaining lifestyle health information online.**

**Theme 1. Process for accessing lifestyle health information online**

- “I guess I wouldn’t go to like Instagram or Facebook to look up health stuff but if it comes up on there, then I might like read it.” [FG1, 17F]
- “I don't really like go on social media for like health info, but when I like scroll on Facebook and I see like news articles or like advice, I just click on the link if I’m interested, and if it's helpful, then I will just keep reading.” [FG1, 18M]
- “I think that the majority of the information I find is accidental...so it’s just stuff that I come across...on social media platforms. Most notably, Instagram because of the explore feature and like its content that is tailored to you.” [FG3, 15F]
- “I feel like for us, like teenagers, there aren’t as many people going on websites ... being regularly would more be like the accounts on Instagram.” [FG5, 15M]
- “I guess for day-to-day info would be social media, but I would trace that if I actually get interested in it and go look at backup research on google or like a research facility who have done research on it.” [FG3, 17M]
- “I think it’s kind of a waste of money, like when you can get the exact same thing for free, and it’s not like I really need it cause I’m like only 15.” [FG5, 15M]
- “I use the health app as well, it kind of just tracks my sleep and steps and everything because I usually have my phone on me wherever I go.” [FG5, 14F]

**Theme 2. Presentation of lifestyle health information online**

- “Some of the websites, they [are] just really convoluted and confusing, so if it’s the first one that pops up but it’s confusing, I will just like go to another one.” [FG1, 18F]
- “I really like the pages that just kind of just sum all the points up like when they speak really, you know, sophisticated, it like, it allows you to trust what they are saying. I also like it when they sum it up at the end in just real simple English so it’s straight to the point.” [FG2, 16F]
- “If there’s like...subheadings within the website or like the answer to it is straightforward rather than in like big paragraphs, because like I wouldn’t be likely to read that and in dot points would be even easier.” [FG3, 17M]
- “Yeah sorry, like headings and it being like scientific, so it is reliable but not um too scientific that we don’t understand what is going on.” [FG3, 18F]
- “A lot of the people who I’m like friends with, they would follow the person as well. And then like, just seeing their content, I kind of go through it, like if they’re professional, you can [kind of] tell, and like they have it in their bios.” [FG5, 14F]
- “The, um, production has to be eye catching for me to actually sort of dig a bit deeper into their account, but otherwise if the content is good, then I’ll follow them.” [FG5, 13F]
- “If they have consistently posted the same sort of content. Not necessarily about the same issue per se but like just if they have got a consistent amount of information like at least 30 posts, if it’s something that I can sort of have a look through.” [FG3, 15F]
- “It’s definitely good if they do have a nice aesthetic side to it, but the content has to be like pretty clear and concise.” [FG5, 13F]

**Theme 3. Credible and reliable lifestyle health information online**

- “Normally, if there is a verified tick or someone like known that’s more trustworthy in that area, in health. So, it’s like, if there is someone new with barely any followers...then it makes it less likely for you to follow that person.” [FG3, 17M]
- “I just go back to the same ones if I find something useful or if it’s helped me, I’ll tend to go back to it because I know it’s reliable and I’ve had a good experience with it.” [FG3, 16M]
- “Usually, it’s sort of based on the quality of the website because usually if it is based... and it looks nice they are sort of putting effort into it, and if it looks dodgy, then it’s probably not going to be as credible.” [FG3, 15F]
- “I just think the ah, the idea of having something government certified, and I think a clear distinction between advice and information.“ [FG4, 17M]
- “It’s quite hard to sift through things that are unbiased, especially on the internet, especially with influencers as well when they are paid.” [FG2, 18F]
- “I also agree with the sponsorship thing because when I found out some of the sponsorships, that’s when I realized that some of this information it wasn’t...like true, it was just because they were being paid to say that.” [FG2, 14F]
- “Another thing that I do sometimes is before I download it, some people leave comments on App Store, which can actually end up being pretty helpful...and then if I think I don’t want it anymore, I just don’t download it.” [FG5, 14F]

**Theme 4. Lifestyle health information relevant to adolescents**

- “I do it because a lot of the times, I feel like the health advice I'm trying to find is when a problem isn't that big of a deal, so I don't think it's worth going to someone like important.” [FG2, 14F]
- “And it’s also like having to see a person in real life is more daunting than actually searching up information for yourself.” [FG3, 17M]
- “I would often go to an influencer even knowing that their information may not be as trustworthy just for the convenience aspect.” [FG5, 15M]
- “I say when you talk about the engaging part, social media really takes the lead there because the type of video they make is captivating and the target audience can always be found.” [FG3, 17M]
- “When it comes to diet information, I don’t really find it helpful because um, because often the diet information is only helpful for like one specific type of group and it’s like, it’s hard to find something that is directed towards me.” [FG2, 14F]
- “So, maybe if there’s a government or some other organization released like a bunch of websites that they think would be really useful and then you could check them out and like maybe get feedback from young people to see which websites they like and things like that, that would definitely help.” [FG4, 16F]

**Theme 5. Perceived behavior changes based on online lifestyle health information**

- “So, in high school, we were always told, oh you have to have 3 meals a day, and I feel like that didn’t really suit my lifestyle, but then when I found this out about intermittent fasting, it just sort of like suited me better than what the government guidelines tell you.” [FG1, 18F]
- “There is this documentary on YouTube called Dominon, and I watched probably only the first 20 minutes, and it’s made me vegan for 2 years so far. It was like, yeah it showed you like a complete other different side to the information that’s on the internet, and so yeah, it kind of did make a big change in my life.” [FG2, 17F]
- “There was this craze about like skipping for fitness, and it was like... I tried it for a bit, and I didn’t really have any results, so I stopped doing it.” [FG3, 17M]
- “I saw some different posts and things like TikTok things on Instagram of bad side effects of drinking dairy, so it was one of the factors that made me not drink dairy anymore.” [FG3, 15F]
- “With most of the things I see, it’s either too hard to integrate for myself or I already do it, so I wouldn’t say that I do anything specific that I’ve seen online.” [FG4, 17F]

## Discussion

### Principal Findings

Overall, this qualitative study provides strong insight from adolescents into the barriers and facilitators of accessing and using lifestyle health information on contemporary digital platforms. To our knowledge, this study is among the first to explore adolescents’ perceptions on their use of these contemporary digital platforms to obtain information and advice about lifestyle health. The results demonstrate that adolescents’ methods for searching for lifestyle health information differ across online platforms, with active searching for information across platforms such as Google and YouTube and passive receiving of information across other social media including Facebook, Instagram, TikTok, and Twitter. Furthermore, adolescents desired information to be well-presented, credible, and relevant to them. The findings from this study can be used to inform future research into the development of effective online lifestyle health promotion strategies and interventions for adolescents.

### Comparison With Prior Work

Previous research has shown that nearly 63% of adolescents use online information broadly to maintain a healthy lifestyle [22]. Many previous studies assessed online information as a whole, without differentiating between online platforms (eg, websites, social media). Our findings from this study appear consistent with previous research regarding the processes that adolescents use to search for health information online; however, this study elicited new findings across the different contemporary digital platforms. From an adolescent perspective, it is apparent why social media is favored over websites when it comes to lifestyle health information. Social media is a common feature in many adolescents’ everyday lives, with mobile devices making access to social media more frequent and personalized [23]. Features available on social media platforms, such as Instagram stories and turning on notification features for favorite accounts, allow content to be highly engaging and of higher production quality. These features increased the perceived credibility of the account by adolescents and allow adolescents to curate who they follow on social media and thereby ensure that the information that they are digesting is relevant to them.

Processes used to search for lifestyle health information by adolescents included searching for information on websites and both actively and passively using social media platforms [17,22]. The passive nature of information exchange on social media is potentially increasing due to the increasing amount of targeted advertising across different social media platforms. In 2021, social media advertising was projected to reach Aus $199 million (US $21.7 million), which is a growth of 4.9% [24]. Users now have less control over the information content within their social media feeds [25]. A study by Hausmann et al [15] suggested only 25% of adolescents agreed that social media could help them obtain useful health information, despite almost ubiquitous use of social media among adolescents. In this study, participants identified several barriers to ascertain whether information on social media was useful. Such barriers included the use of sponsorships and advertising by companies and influencers on social media and the targeted nature of information due to algorithms employed on these platforms. eHealth literacy is the ability to seek, find, evaluate and appraise, integrate, and apply information to solve a health problem in an electronic environment [26]. eHealth literacy was not assessed as part of this study; however, adolescents demonstrated awareness of broad social media advertising and sponsorship strategies. Evaluations of eHealth literacy in the context of contemporary digital platforms warrant further investigation.

Co-design is widely used in the development of eHealth interventions to increase their acceptability and effectiveness among adolescent populations, as they are particularly hard to engage [27,28]. This same methodology can be applied to the development of online health information to increase its appeal in terms of organization of information and relevance to adolescents. A previous systematic review of Australian websites found that very few websites were written specifically for adolescents and none were found to be excellent quality, interactive, and written in plain English [29]. This finding was also demonstrated in our study, with adolescents reporting that information found on websites was often too hard to understand, too difficult navigate, or not relevant to them. To ensure that information is presented in a format that adolescents understand and is relevant to them, co-design of online health information with adolescents could be utilized to increase its acceptability and effectiveness in management of chronic disease risk factors.

The digital space is highly unregulated, and this challenges the credibility of online health information. Due to the rise in user-generated content on digital platforms and popularity of using social media to access lifestyle health information, it is becoming increasingly difficult to regulate digital content, with authors often unidentifiable [30]. As explored in this study, participants reported a preference for “a face behind the account” and being clearly able to see their qualifications in the biography section of their profile to increase credibility and trust in the account. Furthermore, adolescents are acutely aware of advertising and sponsorships within content across social media platforms. The WHO has recognized the influence of food marketing as detrimental to children in many countries. Although regulations have been put in place surrounding advertising to children in Australia (aged 0-14 years) [31], regulations around the world rarely address adolescents [32]. This is despite an increase of more than US $400 million spend on advertising between 2012 and 2019 by the fast food industry targeting children and adolescents [33]. Social media platforms are commercial companies with advertising as their sole generation of income [34]. Currently, there are minimal laws surrounding advertising on social media in Australia, particularly sponsored posts [35]. For example, advertising of weight loss products in Australia must be truthful, accurate, and not mislead consumers [36], but this does not apply to sponsorships. It is important to note that adolescents currently have access to what they perceive as both helpful and harmful lifestyle health information online. Through the addition of regulation and legislation around these areas, there is the capability to make information more useful and credible and potentially lead to behavior change that is helpful for the prevention of chronic disease while also causing minimal harm. Therefore, the regulation of advertising toward adolescents is a challenging space, and further research is required to explore the influence of advertising on contemporary digital platforms toward lifestyle health behaviors of adolescents.

### Strengths and Limitations

This qualitative study has several strengths as well as limitations. We were able to recruit a diverse sample of adolescents to take part in this study, including 41% of participants who spoke a language other than English at home and from different states throughout Australia. Also, this qualitative study is among the first to provide insights into perceived barriers and facilitators of lifestyle health information on contemporary digital platforms, allowing opinions and thoughts to be gathered from the target population. However, it should be emphasized that, as this study was advertised and took place virtually, it may limit the generalizability of the findings to adolescents with higher eHealth literacy skills. For this study, we did not capture data on the eHealth literacy skills of participants. It is possible that groups with lower eHealth literacy may also offer useful insights into their perceptions of online lifestyle health information.

### Recommendations for Development of Online Lifestyle Health Information

Considering the findings from this qualitative study and previous research, a series of recommendations has been developed regarding the development of online lifestyle health information to ensure relevancy, appeal, and engagement for adolescents:

- Employ co-design of lifestyle health information with adolescents for contemporary digital platforms.
- Conduct further research into the regulation of online lifestyle health information for adolescents.
- Consider the eHealth literacy level of adolescents in the development of online lifestyle health information for contemporary digital platforms.

### Conclusions

In summary, this study highlights the abundance and complexity of online lifestyle health information available to adolescents, which is exponentially growing across contemporary digital platforms. Adolescents in this study reported wanting access to information that was credible, appealing, and relevant to them. To develop effective online lifestyle health promotion strategies and interventions, future research should include co-design of information with adolescents and consider their eHealth literacy levels. Furthermore, the influence of advertising on contemporary digital platforms and regulations around this warrants further investigation.

## Appendix

Multimedia Appendix 1 Consolidated criteria for reporting qualitative research (COREQ) checklist.

Multimedia Appendix 2 Supplementary File 2: Focus Group Discussion Guide – Digitalize Study.

## Abbreviations

COREQ: consolidated criteria for reporting qualitative research
WHO: World Health Organization
