# Supplementary material for: Navigating the Online World of Lifestyle Health Information: Qualitative Study With Adolescents
Source: JMIR Pediatr Parent. 2022 Feb 11;5(1):e35165. doi: 10.2196/35165 (PMC8881776; doi:10.2196/35165)
Supplement: Multimedia Appendix 1 [file pediatrics_v5i1e35165_app1.docx]

**Supplementary File 1: Consolidated criteria for reporting qualitative research (COREQ) Checklist**

| **Domain 1: Research team and reflexivity** |  |
| --- | --- |
| *Personal Characteristics* |  |
| 1. Interviewer/facilitator: Which author/s conducted the interview or focus group? | All 5 focus groups were conducted by RR, SJ was note taker, SRP supervised |
| 1. Credentials: What were the researcher’s credentials? E.g. PhD, MD | RR holds a MPH and BBiomedSc |
| 1. Occupation: What was their occupation at the time of the study? | RR is a Research Officer |
| 1. Gender: Was the researcher male or female? | RR is a female |
| *Experience and training* |  |
| 1. What experience or training did the researcher have? | RR has organised, supervised and taken notes for >8 focus groups and conducted >10 individual semi-structured interviews with research participants |
| *Relationship with participants* |  |
| 1. Relationship established: Was a relationship established prior to study commencement? | RR had no prior relationships with any of the adolescents who took part |
| 1. Participant knowledge of the interviewer: What did the participants know about the researcher? e.g. personal goals, reasons for doing the research | None of the participants knew the interviewer prior to the focus groups |
| 1. Interviewer characteristics: What characteristics were reported about the interviewer/facilitator? e.g. Bias, assumptions, reasons and interests in the research topic | No characteristics were reported |
| **Domain 2: study design** |  |
| *Theoretical framework* |  |
| 1. Methodological orientation and Theory: What methodological orientation was stated to underpin the study? e.g. grounded theory, discourse analysis, ethnography, phenomenology, content analysis | Framework approach - thematic analysis |
| *Participant selection* |  |
| 1. Sampling: How were participants selected? e.g. purposive, convenience, consecutive, snowball | Convenience and snowball |
| 1. Method of approach: How were participants approached? e.g. face-to-face, telephone, mail, email | Participants were invited via email and text message |
| 1. Sample size: How many participants were in the study? | 49 invited to take part  37 agreed to take part  32 included in final analysis |
| 1. Non-participation How many people refused to participate or dropped out? Reasons? | 2 declined  10 did not respond  5 agreed to take part but did not attend with no reason given |
| *Setting* |  |
| 1. Setting of data collection: Where was the data collected? e.g. home, clinic, workplace | Online using Zoom teleconference |
| 1. Presence of non-participants: Was anyone else present besides the participants and researchers? | No |
| 1. Description of sample What are the important characteristics of the sample? e.g. demographic data, date Data collection | Demographic data |
| 1. Interview guide: Were questions, prompts, guides provided by the authors? Was it pilot tested? | Questions were asked by the interviewer but not provided to participants. Discussion guide was pilot tested with 2 youth advisors |
| 1. Repeat interviews: Were repeat interviews carried out? If yes, how many? | 5x focus groups were held until thematic saturation was reached |
| 1. Audio/visual recording: Did the research use audio or visual recording to collect the data? | Audio recording was used to collect data via Zoom teleconference and iPhone voice memos |
| 1. Field notes: Were field notes made during and/or after the interview or focus group? | Yes, during |
| 1. Duration: What was the duration of the interviews or focus group? | 45 minutes |
| 1. Data saturation: Was data saturation discussed? | Yes |
| 1. Transcripts returned: Were transcripts returned to participants for comment and/or correction? | No |
| **Domain 3: analysis and findings** |  |
| *Data analysis* |  |
| 1. Number of data coders: How many data coders coded the data? | Two data coders with discrepancies checked by third independent coder |
| 1. Description of the coding tree: Did authors provide a description of the coding tree? | Yes |
| 1. Derivation of themes: Were themes identified in advance or derived from the data? | Derived from the data |
| 1. Software: What software, if applicable, was used to manage the data? | Transcripts produced in Microsoft Word (Version 16.54), thematic analysis in NVivo 12 (12.2.0) |
| 1. Participant checking: Did participants provide feedback on the findings? | No |
| *Reporting* |  |
| 1. Quotations presented: Were participant quotations presented to illustrate the themes / findings? Was each quotation identified? e.g. participant number | Yes |
| 1. Data and findings consistent: Was there consistency between the data presented and the findings? | Yes |
| 1. Clarity of major themes: Were major themes clearly presented in the findings? | Yes |
| 1. Clarity of minor themes: Is there a description of diverse cases or discussion of minor themes? | Yes |
