# Supplementary material for: Navigating the Online World of Lifestyle Health Information: Qualitative Study With Adolescents
Source: JMIR Pediatr Parent. 2022 Feb 11;5(1):e35165. doi: 10.2196/35165 (PMC8881776; doi:10.2196/35165)
Supplement: Multimedia Appendix 2 [file pediatrics_v5i1e35165_app2.docx]

**Supplementary File 2: Focus Group Discussion Guide – Digitalize Study**

**Introductions**

*Moderator to briefly introduce themselves and mention anyone assisting and/or observing the session.*

*Inform the need to audio record the session and remind participants the session will not be video recorded so they can keep their cameras on if they would like to and feel comfortable to do so.*

*Explain that participants can withdraw from the focus group at any time; however, any contribution to the discussion prior to withdrawal cannot be removed from the recording or written transcript.*

Introductions

*Have young people introduce themselves*

*Explain the purpose of the project and bring up respectful discussion*

Main purpose

*Explain purpose of project*

- We are a research group at The University of Sydney conducting a research project to look at how and which digital platforms young people are using to seek lifestyle health-related information online. We also want to understand how helpful these platforms are for health advice
- The group discussion that we will be having will last approximately 60 minutes and we will take short breaks to stretch and check everyone is doing ok.

Respectful discussion

*Explain the importance of everyone contributing respectfully to the discussion, particularly if they feel differently to the person next to them:*

- We will put a link to the participant information sheet in the group chat, please save a copy if you haven’t done so already. There is a list of contacts you can talk to and websites you can access if you feel distressed or upset, if that is what you want to do
- When you feel like you have something to say, please do so. There are many of you in the group and it is important that you all get a chance to express your views.
- Please stay on mute when you are not talking so we can hear everyone clearly.
- There are no right or wrong answers; you do not have to agree with the views of other people in the group.
- You do not have to speak in any particular order.
- While I want to hear what each one of you has to say, it is important that only one person speaks at a time.
- Please keep what is said in this group confidential, by that we mean don’t repeat what is said in the focus group to anyone else
- Please keep your mobile phones on ‘silent mode’
- Does anyone have any questions before we start our group discussion?
- We will check in with everyone at various points during the online interaction to see how everyone is going

*For this discussion, 'lifestyle health and wellbeing' refers to physical activity, nutrition/diet, weight management and sleep. When we refer to ‘online sources’ we are interested in social media platforms, smartphone apps, internet websites and on-demand video/streaming services.*

**Overall**

1. Where do you access health information online?
   1. Prompt: social media platforms, smartphone apps, internet websites and on-demand video/streaming services
2. Why do you seek health information online?
   1. Prompt: Convenience? Do you talk to your GP or other health professionals about this health information? Friends/family? Why/Why not?
3. What type of health content do you find most engaging?
   1. Prompt: diet, physical activity, weight management, sleep etc?
4. How helpful do you feel the health content is that you have accessed online?
   1. Prompt: why/why not?
5. Can you think of a time that you changed something you did (in terms of your health) because of something you saw online?
   1. Prompt: No change or was this a positive or negative change?
6. What would improve your experience of seeking health information online?
   1. Prompt: What content would you like to see more/less? Which platforms? A person behind the account?

***** CHECK IN – MAKE SURE EVERYONE IS OK AND OFFER BREAK OUT ROOMS IF NEEDED*****

*Moderator to proceed with questions related to the 2 platforms most of interest to the group:*

You mentioned <*insert most popular platform e.g., social media*> and <*insert most popular platform e.g., social media*>, let’s chat more about those 2 platforms.

**Social media** 🡪 proceed if top 2 from Q1

1. What social media platforms do you use to look for health information?
   1. Prompt: Instagram, TikTok, Facebook, snapchat
2. What are the reasons behind deciding to follow a particular social media account for health information?
   1. Prompt: regular engagement, new content, size of their existing following, the person?
3. What makes a social media account most appealing to follow for health information?
   1. Prompt: what are the key features i.e., high quality of photos/videos, length of videos, multiple photos, stories, body focused accounts, content topics
4. What makes a social media account least appealing to follow for health information?
   1. Prompt: what are the key features i.e., low quality of photos/videos, no face or person behind the account
5. Do you assess the reliability and usefulness of the account?
   1. Prompt: Credentials, blue tick, celebrity

**Websites** 🡪 proceed if top 2 from Q1

1. How do you search for websites regarding information on lifestyle health information?
   1. Prompt: What search terms do you use? Do you visit same websites regularly?
2. What make health information website most appealing to use?
   1. Prompt: website lay out, information, interactivity, do you find the language of websites hard to understand?
3. What make health information website least appealing to use?
   1. Prompt: website lay out, information, interactivity
4. How did you assess the reliability and usefulness of the health information you have looked up online?
   1. Prompt: How do you decide if a website is trustworthy? Known organization or individual?

***** CHECK IN – MAKE SURE EVERYONE IS OK AND OFFER BREAK OUT ROOMS IF NEEDED*****

**Apps** 🡪 proceed if top 2 from Q1

1. How do you find health information apps to download?
   1. Prompt: word of mouth, app store
2. What make health information apps most appealing to use?
   1. Prompt: subscription fees, features, data ownership
3. What make health information apps least appealing to use?
   1. Prompt: subscription fees, features, data ownership
4. Do you assess the reliability and usefulness of the app?
   1. Prompt: Credentials, number of followers, recommendations from friends etc.?

**Streaming services** 🡪 proceed if top 2 from Q1

1. When you watch lifestyle-related content on streaming services such as Netflix, Stan etc., how do you decide if the information presented to you is trustworthy or not?
   1. Prompt: do you do any further searching on the health topic?
2. What makes lifestyle-related content on streaming services most appealing to watch?
   1. Prompt: quality, presenters
3. What makes lifestyle-related content on streaming services least appealing to watch?
   1. Prompt: quality, presenters

**Conclusions** 🡪 All

***** CHECK IN – MAKE SURE EVERYONE IS OK AND OFFER BREAK OUT ROOMS IF NEEDED*****

1. Are there any final comments you would like to make about the content we have discussed today?

*Many thanks for your time and contribution*
